# P2Y12 inhibitors for the neurointerventionalist

**DOI:** 10.1177/15910199211015042

**Published:** 2021-05-04

**Authors:** Robin J Borchert, Davide Simonato, Charlotte R Hickman, Maurizio Fuschi, Lucie Thibault, Hans Henkes, David Fiorella, Benjamin YQ Tan, Leonard LL Yeo, Hegoda L D Makalanda, Ken Wong, Pervinder Bhogal

**Affiliations:** 1 Department of Clinical Neurosciences, University of Cambridge, Cambridge, UK; 2 Department of Neuroradiology, Oxford University Hospital NHS Foundation Trust, Oxford, UK; 3 Institute of Radiology, University of Padova, Padova, Italy; 4 Addenbrooke’s Hospital, Cambridge University Hospitals NHS Foundation Trust, Cambridge, UK; 5 WFITN Scientific Committee Member, Paris, France; 6 Clinic for Neuroradiology, Klinikum Stuttgart, Stuttgart, Germany; 7 Department of Neurosurgery, Stony Brook University Hospital, Stony Brook, New York, NY, USA; 8 Division of Neurology, National University Health System and Yong Loo Lin School of Medicine, National University of Singapore, Singapore; 9 Royal London Hospital, London, UK

**Keywords:** Antiplatelets, hemorrhage, stroke, aneurysm, thrombectomy

## Abstract

The use of antiplatelets is widespread in clinical practice. However, for neurointerventional procedures, protocols for antiplatelet use are scarce and practice varies between individuals and institutions. This is further complicated by the quantity of antiplatelet agents which differ in route of administration, dosage, onset of action, efficacy and ischemic and hemorrhagic complications. Clarifying the individual characteristics for each antiplatelet agent, and their associated risks, will increasingly become relevant as the practice of mechanical thrombectomy, stenting, coiling and flow diversion procedures grows. The aim of this review is to summarize the existing literature for the use of P2Y12 inhibitors in neurointerventional procedures, examine the quality of the evidence, and highlight areas in need of further research.

## Introduction

The use of antiplatelets in the field of interventional neuroradiology varies significantly between individuals and institutions. This is the result of a lack of randomized control trials (RCTs) and guidelines for the appropriate use of antiplatelets in neurointerventional procedures. Despite this, a wealth of literature exists on this topic and further insights can be drawn from the use of antiplatelets in more established clinical contexts such as percutaneous coronary intervention (PCI). This review focuses on the use of P2Y12 inhibitors in neurointerventional procedures.

**Figure 1.**
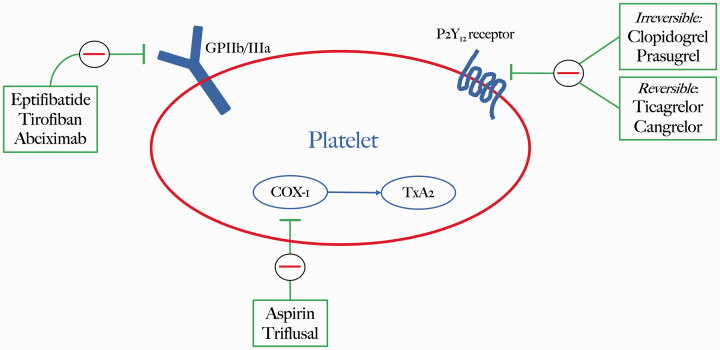
Platelet activation pathways targeted by established and emerging antiplatelet drugs.

Platelets are cell fragments which contribute to primary hemostasis by aggregating and forming the initial plug to arrest bleeding associated with blood vessel wall damage. Platelets are derived from megakaryocytes in bone marrow and their lifespan is around 7–10 days, after which they are removed from the circulation by tissue macrophages. One essential mechanism contributing to platelet aggregation involves the binding of adenosine diphosphate (ADP) to P2Y12 receptors. These P2Y12 receptors are targeted by a variety of antiplatelet drugs in order to reduce the risk of thrombosis (Figure 1).

Clinical trials investigating antiplatelet use in neurointerventional procedures are limited in comparison to other clinical contexts such as secondary prevention of cardiovascular events and PCI. The most appropriate choice of antiplatelet regimens for mechanical thrombectomies, intracranial and extracranial stents, and treatment of cerebral aneurysms, remains unclear. Clinical decision making is further complicated by significant differences between antiplatelet drugs, in terms of route of administration, dosing regimen, onset of action, metabolism, half-life, efficacy and risk of ischemic and hemorrhagic complications. Considering the rapid growth of this field, as well as the multitude of antiplatelet drugs currently available, it is vital to clarify the potential benefits and limitations of each therapy in this clinical setting.

The aim of this review is to provide insight into the existing literature pertaining to the use of antiplatelets in neurointerventional procedures. We use key evidence to provide insight into which antiplatelet drugs are on offer for the neurointerventionalist, their differences in terms of dosing regimen, route of administration and onset/offset of action, as well as the quality of the evidence supporting their use. In some clinical contexts data is lacking and relevant gaps in the literature are also highlighted. This review focuses on P2Y12 inhibitors while Part II discusses GPiib/iiia inhibitors for neurointerventional procedures.^1^

## Methods

A narrative approach was used for this review focusing on identifying the available evidence for the use of antiplatelets in neurointerventional procedures and analysing gaps in the literature. We maintained a broad scope and searched for papers with a combination of key words including “Neurointervention,” “Interventional Neuroradiology,” “Endovascular,” “Thrombectomy,” “Aneurysm,” “Stent,” “Coiling,” “Flow diversion,” “P2Y12,” “Cangrelor,” “Prasugrel,” “Clopidogrel,” “Ticagrelor.” The literature covered in this review was subdivided into each type of P2Y12 inhibitor as well as the relevant type of neurointerventional procedure including thrombectomy, intra- and extracranial stenting, coiling, stent-assisted coiling and flow diversion. We presented either the highest quality studies (i.e. RCTs or core-lab audited registries) or the largest sample sizes if such studies were not available. Case reports, and small series of less than five patients were excluded. Considering the rapid progression of this field in terms of antiplatelet agents on offer, neurointerventional techniques and standards of care, the timeframe for studies included in this review was 2000–2021 (inclusive). Dosing regimens for each P2Y12 inhibitor described in Table 1 reflect those used in the highest quality studies identified for the respective antiplatelet agent. The cardiac literature discussed in this review includes large-scale RCTs which have influenced practice and provide insight into antiplatelet use for neurointerventional procedures.

**Table 1.** Classification, route, dose, onset of action and half-life for P2Y12 inhibitor antiplatelet therapies in neurointerventional procedures.

	Mechanism	Route	Loading/bolus dose	Maintenance dose	Onset of action	Half-life
Clopidogrel^1,35^	P2Y12 inhibitor	Oral	600 mg	75 mg OD	2 h	30 min (active metabolite)
Ticagrelor^4,34–36^,^95^	P2Y12 inhibitor	Oral	180 mg	60–90 mg BD	30 min–4 h	8–12 h (active metabolite)
Prasugrel^4,6^,^53^,^62^	P2Y12 inhibitor	Oral	20–60 mg	5–10 mg OD	30 min–4 h	30–60 min (distribution half-life of active metabolite)
Cangrelor^4,70^,^77^,^96^	P2Y12 inhibitor	IV	30 µg/kg	4 µg/kg/min	2 min	3–6 min

Onset of action is described as time to onset, percentage platelet inhibition or peak plasma concentration depending on the existing literature. These are common dosing regimens described in the literature; please refer to local guidelines, policies and clinical expertise to guide clinical practice.

## Clopidogrel

### Mechanism, pharmacokinetics and general considerations

Clopidogrel is a pro-drug that is rapidly absorbed in the gastrointestinal system following oral administration.^2^ Most of the drug is transformed into an inactive metabolite but the remaining pro-drug is converted to the active form, thiol metabolite, which irreversibly inhibits the P2Y12 receptor ADP-binding site, thereby inhibiting platelet aggregation.^3^ The onset of action following an oral loading dose of clopidogrel is around 2 h while platelet activity is typically recovered within 5–10 days following cessation^4^ (Table 1). However, the pharmacokinetics of clopidogrel vary significantly between individuals which warrants caution when used peri-procedurally.^5^

Responsiveness to clopidogrel and the half-life of its active metabolite can be influenced by genetic polymorphisms, diet, smoking, alcohol and demographics.^5^ There is a lack of homogeneity within the literature for defining clopidogrel responsiveness, making it difficult to determine the optimal P2Y12 reference range. For example, one study may define clopidogrel response as >20% inhibition, while others use >30% or >40% inhibition as their threshold.^6,7^

Specific patient populations have been shown to be less responsive to clopidogrel. For example, clopidogrel is less effective for secondary prevention following stroke in patients with CYP2C19 loss-of-function alleles which is more common in Asian populations.^8,9^ In the cardiology literature, using a CYP2C19 genotype-guided approach improves patient outcomes following PCI by avoiding the administration of clopidogrel to patients carrying one or both loss-of-function alleles (up to 35% of the US population is estimated to have reduced response to clopidogrel).^10,11^ This approach was non-inferior to standard treatment with ticagrelor or prasugrel during primary PCI with respect to thrombotic events, and was associated with lower incidence of bleeding.^10^ Patients with type 2 diabetes mellitus undergoing interventional procedures also have a diminished response to clopidogrel.^12^

Overall, reduced responsiveness to clopidogrel is common and should be considered in the context of neurointerventional procedures. Prasugrel or ticagrelor can be considered as alternatives in these situations (discussed in the relevant sections below).

### Clopidogrel for thrombectomy and stenting procedures

The literature covering peri-procedural use of clopidogrel for mechanical thrombectomy and stenting is limited. Following the success of the MR CLEAN trial ^13^ which demonstrated the benefit of mechanical thrombectomy for acute ischemic stroke, a subgroup analysis was performed which found that pre-procedural treatment with antiplatelet agents did not alter the success of intra-arterial treatment.^14^ However, in those with successful reperfusion, prior antiplatelet treatment was associated with better functional outcomes, but a higher risk of intracranial hemorrhage. Hemorrhagic risk was 15% in those with pre-procedural antiplatelet use compared to 4% without. Although this analysis was not sufficiently powered to distinguish the individual effects of different antiplatelets such as clopidogrel, or specific dual antiplatelet regimens, it highlighted the importance of balancing the potential benefits versus risks associated with antiplatelets peri-procedurally.

Since the MR CLEAN trial, and contradictory to its subgroup analysis findings, Pandhi et al. found that pre-procedural treatment with antiplatelets increased the likelihood of successful reperfusion without affecting the risk of symptomatic intracranial hemorrhage or functional outcomes at three months.^15^ Nonetheless this study was also limited in that it was a retrospective design with a small cohort (only 14 patients were on clopidogrel mono- or dual-antiplatelet therapy) and included multiple variations of antiplatelet regimens and agents.

For tandem occlusions treated with stenting and mechanical thrombectomy, peri-procedural use of antiplatelets is often considered to reduce the risk of in-stent thrombosis and re-occlusion but can also increase the risk of intracranial hemorrhage.^16^ IV administration of aspirin or a GPIIb/IIIa agent, such as eptifibatide or tirofiban, can be considered and this can be switched to clopidogrel post-procedurally.^17,18^ However, there is heterogeneity in practice and a lack of reliable high-quality evidence to make specific antiplatelet recommendations for this clinical context at present.

Overall, the evidence supporting the peri-procedural use of clopidogrel for thrombectomy and stenting procedures is limited, and there is a significant gap in the current literature regarding the benefits and potential hemorrhagic complications associated with the drug. These contradictions will hopefully be addressed in an ongoing large-scale RCT (MR CLEAN-MED, ISRCTN 76741621) which is investigating peri-procedural antithrombotic treatments for mechanical thrombectomy.

### Clopidogrel for cerebral aneurysm procedures

Interventional procedures for intracranial aneurysms such as coil embolization and flow-diversion can benefit from antiplatelets like clopidogrel by reducing the risk of thromboembolic complications.

In a prospective study of 63 patients undergoing aneurysm coiling, pre-procedural clopidogrel was not associated with any hemorrhagic complications, and the thromboembolic complications were lower (3.2%) when compared to aspirin (7.2%).^19^ However, since this study, it has become evident that stratifying patients based on VerifyNow assays and calculating Platelet Reactivity Unit (PRU) values reveals that responsiveness to clopidogrel is an important predictor of complications. Both hyper- and hypo-responders to clopidogrel undergoing coil embolization or flow diversion for intracranial aneurysms have an increased risk of thromboembolic and hemorrhagic complications.^20–25^

This may be related to heterogeneity in patient response, for example a 5 mg dose of clopidogrel in a hyper-responder had a similar effect to a 75 mg dose in a normal responder.^26^ Resistance to clopidogrel is also relatively common in these patient cohorts highlighting the importance of evaluating and titrating pre-procedural treatment with clopidogrel.^20,25^ One RCT used this approach and found that modifying the pre-procedural antiplatelet regimen in hypo-responders reduced the risk of thromboembolic events following coiling of unruptured aneurysms.^27^

The significance of hypo- and hyper-response to pre-procedural clopidogrel was demonstrated in the context of Pipeline Embolization Devices (PED) as well; both high and low PRU values were associated with increased thromboembolic and hemorrhagic complications in unruptured ^28^ and ruptured aneurysms.^29^ Hemorrhagic complications were more common in high responders (PRU values <70) while thromboembolic complications were more common in low responders (PRU values >150). Nonetheless, even when clopidogrel doses were adjusted according to PRU values, in-stent thrombosis ranged from 3.5% to 16%.^30,31^ Nuisance bleeding (easy bruising, petechia, ecchymosis, etc.) has also proven problematic when using antiplatelet regimens post-procedurally following PED deployment, although the incidence (27.3% of patients) was similar across different antiplatelet regimens.^32^

These studies highlight the limitations of using clopidogrel peri-procedurally and emphasize the importance of accounting for clopidogrel responsiveness when considering the use of clopidogrel as a pre-medication for flow diversion or coil embolization. The current literature suggests that one approach is to take PRU values into account to ensure adequate dosing, or to consider alternative antiplatelet agents which have less heterogeneity in patient response. Further RCTs comparing antiplatelet agents, regimens, dosing and outcomes for treated intracranial aneurysms are indicated in order to update protocols and ensure best practice.

## Ticagrelor

### Mechanism, pharmacokinetics and general considerations

Ticagrelor is another oral P2Y12 receptor inhibitor which, unlike clopidogrel and prasugrel, *reversibly* binds the P2Y12 receptor. Ticagrelor also has a faster onset of platelet inhibition compared to clopidogrel.^26^ Ticagrelor’s onset of action is ∼30 min and 80–90% platelet inhibition is achieved within 2–4 h.^33,34^ In the context of neurointerventional procedures, a common dosing regimen includes 180 mg loading dose of ticagrelor and/or a 60–90 mg twice a day maintenance dose.^34,35^

An advantage of ticagrelor for the neurointerventionalist is its rapid onset of action.^36^ However, there are concerns surrounding an increased risk of hemorrhage associated with this agent (evidence discussed below) and platelet transfusions may also be less effective for the reversal of ticagrelor.^37^ Another disadvantage is that dyspnoea is a common side effect of this drug.^38^ Unfortunately, the evidence in support of ticagrelor use in neurointerventional procedures is scarce, and mainly limited to retrospective studies and case series, although RCTs and large registries are anticipated.

### Ticagrelor for thrombectomy and stenting procedures

Multiple large scale RCTs, both past and present, have investigated ticagrelor versus other antiplatelet agents in stroke and transient ischemic attack (TIA). However, mechanical thrombectomy and stenting is consistently used as an exclusion criteria.^39–41^

In a retrospective study, a loading dose of ticagrelor prior to mechanical thrombectomy for anterior circulation stroke increased the risk of symptomatic intracranial hemorrhage compared to clopidogrel.^42^ There were no differences found in functional outcome or mortality rates between the two drugs, but ticagrelor may be less effective than clopidogrel for secondary prevention of stroke and TIA.^43^

With regards to extracranial procedures, in a series of patients with carotid stenosis who underwent angioplasty and stenting, clopidogrel resistant patients who received ticagrelor 180 mg pre-procedurally had similar rates of ischemic and hemorrhagic complications compared to those who received the standard 300 mg clopidogrel dose.^44^ The authors did note that at the pre-specified one-year follow-up angiogram, the rate of in-stent re-stenosis was significantly lower in the patients treated with ticagrelor (0% vs 4% for clopidogrel, P=0.03).

Pre-procedural ticagrelor 180 mg for acute carotid artery stenting and mechanical thrombectomy for tandem occlusions has also been described.^45^ However, this was a retrospective study and not powered sufficiently to compare outcomes and complications between the different antiplatelet agents and regimens used. Furthermore, there is an ongoing multi-center RCT Ticagrelor Versus Clopidogrel in Carotid Artery Stenting (PRECISE-MRI), which should shed light on the utility of ticagrelor, compared to clopidogrel, for extracranial stenting.^46^ Overall, high quality data pertaining to the use of ticagrelor for thrombectomy and stenting procedures is lacking, making it difficult to provide evidence-based recommendations for practice.

### Ticagrelor for cerebral aneurysm procedures

Ticagrelor has been used pre-procedurally for coiling and flow diversion of intracranial aneurysms. The findings of two retrospective cohort studies suggested that pre-procedural dual antiplatelet therapy with ticagrelor and aspirin is a safe alternative, with similar rates of thromboembolic complications to other regimens for both flow diversion and stent-assisted coiling of unruptured intracranial aneurysms.^47,48^ One protocol involved administration of ticagrelor 180 mg the night before the procedure, a second 180 mg loading dose pre-procedurally, and IV aspirin 250 mg at the start of the procedure.^48^ The other study provided only limited information on the dosing regimen adopted.^47^ The use of ticagrelor may also be of particular relevance and benefit to patients undergoing coiling or flow diversion who are non-responders to clopidogrel.^49^

More recent studies support these findings; ticagrelor monotherapy for Pipeline flow diverter deployment in ruptured and unruptured aneurysms was effective with low rates of in-stent thrombosis (8.4%) and re-bleeding (4.2%).^50^ However, this was a small retrospective study (*n* = 24 patients) and requires further validation in larger cohorts. A larger meta-analysis investigating antiplatelet regimens for flow diverter use found that the risk of ischemic and hemorrhagic complications of ticagrelor-based dual antiplatelet therapy was not significantly different compared to clopidogrel-based regimens but was associated with reduced mortality.^51^ Nonetheless, the effect was largely driven by a single study, and after correction for this study, the statistical significance was lost. This further highlights the need for more reliable evidence to inform the use of ticagrelor for the treatment of cerebral aneurysms.

## Prasugrel

### Mechanism, pharmacokinetics and general considerations

Prasugrel, another P2Y12 receptor inhibitor, has a similar mechanism of action to clopidogrel. Advantages of prasugrel include a faster onset of action and less variability in patient response. Onset of action is between 15 and 30 min with peak plasma concentration for prasugrel’s active metabolite, R-138727, achieved at ∼30 min^34,52^ (Table 1).

A common dosing regimen of prasugrel for interventional procedures consists of a 20–60 mg loading dose and/or a 5–10 mg daily maintenance dose.^6,34,53^ Following administration of 40–60 mg of prasugrel, 75–80% platelet inhibition is achieved at 4 h and pre-treatment platelet activity is recovered in most patients within seven-days following cessation.^54^

### Prasugrel for thrombectomy and stenting procedures

There is a lack of data on the use of prasugrel in thrombectomy and stenting although the available evidence suggests it may be associated with increased hemorrhagic complications. One retrospective cohort study investigated 76 patients undergoing a variety of neurointerventional procedures, including aneurysm coiling and flow diversion, as well as intra- and extracranial stenting. The aspirin (325 mg pre- and post-procedure) and prasugrel (60 mg pre-procedure, 10 mg daily post-procedure) regimen in clopidogrel-resistant patients was associated with a significantly higher rate of hemorrhagic complications compared to the aspirin and clopidogrel (75 mg pre- and post-procedure) regimen (19.4% vs. 3.6%, *P* = 0.02).^6^

The increased bleeding risk is also reflected in the cardiovascular literature. The TRITON-TIMI 38 trial compared prasugrel (60 mg loading dose, 10 mg maintenance dose) to clopidogrel (300 mg loading dose, 75 mg daily dose) in 13,608 patients scheduled to undergo PCI with moderate-high risk acute coronary syndrome.^55,56^ Prasugrel was associated with a significantly reduced rate of death and in-stent thrombosis, with increased rates of major bleeding compared to clopidogrel. It was also highlighted that patients with a history of cerebrovascular events, who were <60 kg, or over 75 years of age, had no net benefit from prasugrel. Nuisance bleeding while on prasugrel was also relatively common (13.6%) and associated with high rates of discontinuation.^57^

In the context of secondary prevention of ischemic stroke, prasugrel was demonstrated to be non-inferior to clopidogrel with similar rates of bleeding. This suggests prasugrel may be beneficial to patients who respond poorly to clopidogrel.^58^ Whether these insights from other specialties are translatable to neurointerventional procedures is not yet clear, however the existing evidence suggests caution should be taken with prasugrel due to the risks of bleeding compared to more conventional antiplatelet agents.

### Prasugrel for cerebral aneurysm procedures

Administration of prasugrel for neurointervention involving potentially hemorrhagic procedures has been described in various retrospective and prospective cohort studies, as well as meta-analyses.

In a prospective study of 222 unruptured aneurysms that underwent endovascular treatment (including coiling, stent-assisted coiling and flow diversion), patients either received low-dose prasugrel (20 or 30 mg) or clopidogrel (300 mg) pre-procedurally. Prasugrel was associated with more effective platelet inhibition than clopidogrel (60.2% vs. 22.1%, respectively) with no difference in thromboembolic or hemorrhagic complications.^59^ When these patients were followed up for a mean of 1.5 (range 1–7) months, no thromboembolic or hemorrhagic complications were reported.^60^ These findings were supported by a smaller retrospective study comparing prasugrel to clopidogrel in neurointerventional procedures.^61^

For stent-assisted endovascular coil embolization of unruptured aneurysms, a separate retrospective study included 207 patients receiving low-dose (20 mg) prasugrel and 90 patients receiving standard dose (75 mg) clopidogrel.^62^ Thromboembolic events occurred less frequently with prasugrel compared to clopidogrel (0.9% vs. 6.4%; *P* = 0.01). Pre-medication with clopidogrel was the only variable significantly associated with thromboembolic complications (OR = 13.200; 95% CI 1.476–118.025; *P* = 0.021). These findings were echoed in a similar retrospective study in France which compared pre-medication with aspirin (75 mg) + clopidogrel (75 mg) versus aspirin (75 mg) + prasugrel (60 mg) for stent-assisted coiling of unruptured intracranial aneurysms.^63^ There were no significant differences in hemorrhagic complications, but the clopidogrel regimen was associated with a higher number of thromboembolic complications and in-stent thrombosis as well as increased morbidity (measured with mRS) at 30 days.

Prasugrel may also benefit procedures involving flow diversion. A recent retrospective study administered prasugrel (30 mg) and aspirin (300 mg) pre-procedurally followed by a prasugrel (10 mg) maintenance dose in patients with intracranial aneurysms treated with flow diverters.^64^ This regimen achieved effective platelet inhibition with few hemorrhagic (1.4%) or thromboembolic (4.8%) complications. At six-month follow-up, angiography demonstrated a 95.4% aneurysm occlusion rate supporting the feasibility of using low-dose prasugrel peri-procedurally for flow diversion interventions.

Compared to clopidogrel, there are a number of advantages of prasugrel in the context of Pipeline flow diverters.^65^ Patients with low response to clopidogrel were pre-medicated with prasugrel (40–60 mg) and aspirin (81 mg) followed by prasugrel 10 mg for pipeline flow diversion of a cerebral aneurysm. This was associated with a lower incidence of thromboembolic and hemorrhagic complications, as well as mortality, compared to the clopidogrel treated group. Although this study did not reach statistical significance, it highlighted that prasugrel is likely a safe alternative for clopidogrel resistant patients undergoing flow diversion.

A meta-analysis of the prospective and retrospective studies reviewed here found that low-dose prasugrel (20 mg loading with 5 mg maintenance) administered one day prior to treatment of an unruptured intracranial aneurysm was associated with a significant reduction in treatment-related complications (OR = 0.36; 95% CI, 0.17–74, *P* = 0.006) compared to standard-dose clopidogrel (300 mg loading with 75 mg maintenance).^66^ However, high-dose prasugrel (60 mg loading with 10 mg maintenance) was associated with significantly higher peri-procedural and early (within 24 h) hemorrhagic events compared to low-dose (20 mg loading with 5 mg maintenance) prasugrel (9.3% vs. 0.6% respectively). These conclusions, which highlight the potential benefit of prasugrel, were also supported in two other recent meta-analyses.^51,67^

In summary, early evidence suggests that low-dose prasugrel has advantages which include fast onset of action, more efficient platelet inhibition than clopidogrel, and restoration of platelet activity 24 h after cessation. The literature reviewed here proposes that prasugrel is likely a safe alternative for peri-procedural antiplatelet treatment in coiling and flow diversion for intracranial aneurysms, although most of the evidence to support this is retrospective in nature. For ischemic procedures such as thrombectomy, the evidence is less clear with only a limited number of studies and high rates of hemorrhagic complications, which may be the consequence of using high-dose prasugrel. Concerns surrounding prasugrel’s potential hemorrhagic risks are further highlighted by the US Food and Drug administration’s “black box” warning for increased risk of bleeding, particularly in the context of patients with previous stroke or TIA. Further evidence in the form of prospective investigations will be required to clarify this.

## Cangrelor

### Mechanism, pharmacokinetics and general considerations

Cangrelor is a unique P2Y12 receptor inhibitor in that it can be administered intravenously. Cangrelor *reversibly* inhibits the platelet ADP P2Y12 pathway in a dose-dependent manner achieving near-complete (>90%) inhibition of platelet aggregation.^4,68^

Cangrelor has many advantages due to its superior pharmacokinetic profile compared to clopidogrel, such as rapid onset of action (∼2 min), high degree of platelet inhibition, short half-life with recovery of platelet activity at ∼60 min following cessation, and easier quantification of action.^4^ However, switching between cangrelor and oral antiplatelets can be challenging. Preliminary recommendations, not specific to neurointervention, include starting IV cangrelor three to four days following prasugrel discontinuation or two to three days following clopidogrel or ticagrelor discontinuation.^4^ Prasugrel and clopidogrel can be re-started immediately after cangrelor discontinuation.^4^

Due to its route of administration and potency, cangrelor has a theoretical increased risk of bleeding compared to oral antiplatelet agents, and in many countries cangrelor is expensive compared to oral antiplatelets. The literature covering cangrelor use in neurointerventional procedures is very limited, particularly for treatment of cerebral aneurysms.

### Cangrelor for thrombectomy and stenting procedures

Considering its relatively recent introduction to clinical practice, there have been no large-scale trials of cangrelor in acute ischemic stroke patients thus far. In a retrospective series, 37 patients who underwent carotid artery or intracranial stent placement were administered a bolus dose of cangrelor (5 mcg/kg) and maintenance infusion (0.75–1 mcg/kg/min) which was titrated to reach the target range of 50–150 PRU.^69^ Two patients had post-procedural thromboembolic complications but no hemorrhagic events occurred. This is noteworthy as it was the first series describing a cangrelor regimen for neurointerventional stenting. In another small study of 10 patients who underwent extracranial stenting, stent-assisted coiling or flow diversion, a stronger regimen was used with a 30 mcg/kg IV bolus of cangrelor, and 4 mcg/kg/min maintenance infusion followed by 81 mg aspirin and 180 mg ticagrelor post-procedure.^70^ All procedures were successful with recanalization of occluded arteries and occlusion of aneurysms with one thrombotic and three new, or ongoing, hemorrhagic complications. Additional small-scale studies investigating cangrelor for stenting in acute ischemic stroke have also yielded positive outcomes, albeit with varying cangrelor dosing regimens.^71–73^ Despite the differences in cangrelor dosing, these small cohorts have all demonstrated that IV cangrelor may be a viable option in acute neurointerventional procedures although a widely-accepted dosing protocol has yet to be developed.

Insight can also be gained from the PCI literature where cangrelor has been studied in-depth. In the CHAMPION PHOENIX trial^74–76^ of >11,000 patients undergoing urgent or elective PCI, cangrelor reduced the risk of death, MI, stent thrombosis and ischemia-driven revascularization (4.7% compared to 5.9% for clopidogrel).^75^ Cangrelor was associated with lower rates of stent thrombosis (0.8%) relative to clopidogrel (1.4%) with no increase in severe bleeding.^74–76^

Considering the advantages of prasugrel seen in the PCI literature as well as the initial studies using it for neurointervention, cangrelor is a promising antiplatelet agent for thrombectomy and stenting but will require higher quality evidence to confirm its benefits and appropriate dosing regimen.

### Cangrelor for cerebral aneurysm procedures

Few studies have investigated pre-medication with cangrelor for aneurysm treatment and those that have included <10 patients. This may be explained by the obvious advantages of an intravenous, fast-acting antiplatelet agent for acute procedures which are less relevant to planned elective procedures for cerebral aneurysms. Nonetheless, a case series (*n* = 7) of patients undergoing stent-assisted coiling or flow diversion embolization of challenging ruptured and unruptured intracranial aneurysms, found that cangrelor was a viable option as a peri-procedural antiplatelet with one hemorrhagic complication.^77^ Further investigations will certainly be needed though to support the use of cangrelor in the context of intracerebral aneurysms.

## Aspirin and P2Y12 inhibitors

The important role of aspirin for neurointerventional procedures must be acknowledged when considering the use of P2Y12 agents. Aspirin’s wide-scale adoption can in part be attributed to its use in the early, but crucial, RCTs demonstrating its benefits, particularly in patients at increased risk of occlusive vascular events like ischemic stroke.^78–80^ More specifically to neurointervention, early evidence highlighted that medical management with antiplatelet agents like aspirin may be superior to neurointerventional management in clinical contexts such as intracranial arterial sclerosis.^81^ These studies established aspirin as one of the Gold Standard treatments for vaso-occlusive pathologies. As a result, it is often used as a control to which other antiplatelet regimens are compared, or constitutes the second agent in combination with a P2Y12 inhibitor.^39,41,82^

Looking at clopidogrel specifically, most of the neurointerventional literature uses dual antiplatelet regimens together with aspirin. For example, in studies investigating peri-procedural use of antiplatelets for mechanical thrombectomy, clopidogrel administration is usually combined with aspirin, including in the pivotal Mr CLEAN trial.^14,15^ In the study of endovascular treatment of tandem occlusions discussed earlier, clopidogrel (600 mg oral) was used in conjunction with aspirin (650 mg oral), not as a monotherapy.^45^

Similarly, in a large retrospective study of mechanical thrombectomy and extracranial stenting for ischemic stroke caused by intracranial tandem occlusions, aspirin (500 mg IV) was combined with either clopidogrel (600 mg oral) or ticagrelor (180 mg oral).^42^ Prasugrel and cangrelor have also been combined with aspirin (325 mg oral for seven days prior to procedure and 81 mg oral, respectively) for neurointerventional procedures.^6,70^ With respect to neurointerventional management of intracranial aneurysms, a large systematic review of flow diversion procedures found that when a P2Y12 agent was used, such as clopidogrel, ticagrelor or prasugrel, they were often administered together with aspirin.^51^ These dosing regimens included clopidogrel (75 mg oral) plus aspirin (325 mg oral),^28^ ticagrelor (180 mg oral) plus aspirin (250 mg IV)^48^ or prasugrel (60 mg oral) plus aspirin (75 mg oral).^63^

Although aspirin is commonly used in combination with P2Y12 inhibitors for various interventional procedures, significant heterogeneity in dosing, timing and route of access still exists. This adds another level of complexity when delineating the thromboembolic and hemorrhagic risks/benefits of individual P2Y12 agents across neurointerventional procedures and further emphasizes the need for high quality prospective studies to identify the regimens which are most beneficial for patients. Nonetheless, the literature on P2Y12 inhibitors to date has relied heavily on combining these agents with aspirin, and in practice, this combination should be considered when making clinical decisions regarding peri-procedural antiplatelet therapy.

## Platelet and genetic testing

Patients can vary in their response to antiplatelet agents, like clopidogrel, which are known to rely on metabolic pathways and genetic variants. These can vary significantly across the population.^10,11^ Patients may be hypo- or hyper-responders to an antiplatelet drug, which can lead to increased risk of hemorrhagic and thromboembolic complications.^28^ One approach to addressing this heterogeneity is with platelet function testing; for example, the VerifyNow assay can estimate the extent of P2Y12 inhibition.^83^ This degree of inhibition is reported in PRU with higher values associated with reduced platelet inhibition which would be seen in a hypo-responder to an antiplatelet such as clopidogrel.

In practice, the utility of platelet function testing is debated. Potential advantages for patients who demonstrate hypo- or hyper-response to a P2Y12 inhibitor include adjusting antiplatelet dosing to achieve PRU values within a target range,^26^ or switching these individuals to an alternative antiplatelet agent.^27,59,60^ The benefits of these approaches are often unclear. For example, in one study investigating PED for intracranial aneurysms, adjusting the antiplatelet regimen to achieve platelet inhibition within a target range resulted in a high rate of in-pipeline stenosis^84^ similar to that reported in other studies which did not adjust their antiplatelet regimen according to platelet function testing.^85,86^ Although adjusting platelet function to an optimal range should theoretically reduce hemorrhagic and/or thromboembolic complications, this has been difficult to demonstrate in practice and accounts for the lack of platelet function testing at many neurointerventional centers.

Similarly, a growing body of evidence suggests that genetic differences influence response to antiplatelet medications. This is of particular relevance to clopidogrel; around 30% of patients do not respond to clopidogrel appropriately as measured by PRU testing,^87^ and this could be related to genetic variation in the cytochrome P450 isoenzymes regulated by alleles such as CYP2C19 and CYP2C9.^88^ Loss of function genotypes for these alleles do not alter the pharmacodynamics of other P2Y12 inhibitors such as prasugrel.^88^ This raises the question if genetic testing for common cytochrome P450 loss-of-function alleles should be incorporated into everyday practice. In the cardiovascular literature, patient outcomes following PCI improve when a CYP2C19 genotype approach to antiplatelet therapy is used.^11^ However, in the field of interventional neuroradiology, the number of studies investigating the association between genetic polymorphisms and outcomes is limited. Preliminary evidence suggests that for patients receiving clopidogrel peri-procedurally for stent-assisted coiling of intracranial aneurysms or carotid stenting, CYP2C19 variants are associated with increased risk of bleeding and cerebral ischemic events.^89–93^ Some neurointerventionalists have already recommended genetic testing for patients undergoing neurointerventional procedures.^94^ However, implementation of this has not yet been widely adopted. Future prospective studies, similar to what has been achieved in the field of cardiology and PCI, will help inform practice and determine if adjusting antiplatelet regimens according to genotypic variants will benefit patients.

## Limitations

The application of antiplatelet agents to neurointerventional procedures and its supporting evidence is still in the early stages. As a result, using a narrative approach provides the opportunity to investigate the types of evidence available with a wide scope as well as identify trends and gaps in the existing literature. However, this approach has its limitations such as unintentionally overlooking relevant studies which otherwise may have been covered in a systematic review, but with a more limited scope. This review has other limitations as well; considering the large gamut of antiplatelet agents, dosing regimens, patient populations and procedures covered here, there is likely some heterogeneity in patient and disease characteristics between papers. These characteristics, which vary between studies, are often not reported, for example aneurysm size, ruptured versus unruptured state of the aneurysms and different length of follow-up periods for outcomes. We have clarified these aspects where possible, but inevitably these differences may result in unequal comparisons and conclusions drawn between antiplatelet agents, and their applications to neurointerventional procedures. Finally, as this is a rapidly progressing field, the time points used in older antiplatelet studies, particularly for clopidogrel, may have a different reference for standard of care compared to newer studies using novel interventional devices. Nonetheless, we have aimed to make this review an accurate, up-to-date depiction of antiplatelet agents and their risk profiles currently available to neurointerventionalists.

## Conclusion

With an armoury of antiplatelet agents available, the most appropriate and practical approach to antiplatelet use in neurointerventional procedures can often be unclear. The existing literature summarized here, drawn from neurointerventional studies and other fields where antiplatelets play a prominent role, can provide insight into how these agents have been applied so far and identify areas where further research is needed. Additionally, new agents and medical implant devices with surface modifications are now in development and could show promising therapeutic effects. Ongoing and future large-scale RCTs are needed, and will likely shed further light on recommended antiplatelet regimens for the neurointerventionalist.
